# Special Issue: Advances of Ionic Liquids in Organic, Polymer and Material Chemistry: Key Trends in Green Chemistry

**DOI:** 10.3390/molecules31162852

**Published:** 2026-08-15

**Authors:** Pradip K. Bhowmik, Biplab Banerjee

**Affiliations:** 1 Department of Chemistry and Biochemistry, University of Nevada Las Vegas, 4505 S. Maryland Parkway Box 454003, Las Vegas, NV 89154-4003, USA; 2 Department of Chemistry, Central University of Punjab, Bathinda 151001, Punjab, India; biplab.banerjee@cup.edu.in

Ionic liquids (ILs) are a class of solvents that have been extensively explored for several decades as promising materials for various applications and continue to be explored due to their tunable physicochemical properties. The physicochemical properties include low melting transitions, a wide liquid range, wide electrochemical windows, viscosity, high density, low surface tension, low vapor pressure, low flammability, wide-range solubility, highly polar yet noncoordinating solvents, and nonvolatility. They can interact with diverse materials through a multitude of interactions, including electrostatic, dispersive, hydrogen bonding, π–π, and dipolar interactions afforded by their readily tunable chemical structures of cations and anions. They are often referred to as designer solvents because most of their physicochemical properties can be tuned by the sheer combination of different cations and anions. They find increasing use across various disciplines, including organic chemistry, polymer chemistry, materials chemistry, inorganic chemistry, analytical chemistry, biotechnology, and medicine. By incorporating certain functional groups into their chemical structures, relatively new subclasses of ILs have emerged in recent years, including polymeric ionic liquids (PILs), magnetic ionic liquids (MILs), zwitterionic ionic liquids (ZILs), dicationic or dianionic liquids (DILs), chiral ionic liquids (CILs), redox-active ionic liquids (RAILs), and fluorescent ionic liquids (FILs). Although there are numerous reviews and books that appeared in the literature on the synthesis, properties, and applications over several decades, it is worthwhile to mention several recent reviews [1,2,3,4,5,6,7,8,9,10,11] on the topic so that trends in the field can be judged. In short, these reviews have detailed applications in drug delivery, biomedical fields [2,8,9], nanoparticle synthesis and organic synthesis [1,7], lubricants [1], biomolecule purifications [4], separations [4], and energy-related applications, including batteries and supercapacitors, fuel cells, dye-sensitized solar cells, and perovskite solar cells [1,3,5,6,10]. Deep eutectic solvents (DESs) are also an emerging class of mixtures of components that are characterized by significant depression in melting points when compared to those of constituent components. They are promising for application as inexpensive designer solvents exhibiting a unique suite of tunable physicochemical properties. They typically can be prepared from the combination of quaternary ammonium salts and metal chlorides or metal chloride hydrates or hydrogen bond donors (HBDs) such as amides, carboxylic acids, and polyols. They can also be synthesized from the combination of metal chloride hydrates and HBDs as well as nonionic versions made of hydrogen bond acceptors (HBAs) and HBDs. In brief, DESs promise superior performances over traditional ILs. They are easy to prepare in high purity without any purification steps and with a 100% atom economy. Noteworthy is the extremely low cost and the large availability of many precursors as bulk chemicals, which make DESs prepared at much lower costs than ILs and potentially useful for large-scale applications. They have gained a lot of attractions, since they share many of the advantageous properties of ILs, namely negligible vapor pressure, good thermal and chemical stability, non-flammability, and potential as tunable solvents. They are multi-component systems as opposed to ILs [11].

This Special Issue, titled “Advances of Ionic Liquids in Organic, Polymer and Material Chemistry: Key Trends in Green Chemistry”, aims to collect 16 research articles of outstanding research groups working with ionic liquids and DESs to shed light on the recent activities in this important field. Interestingly enough, we have also collected six review articles in this Special Issue that certainly demonstrated the ongoing vigorous activities of the ILs and associated ever-increasing new classes of ILs. The articles include many aspects of ILs and DESs ranging from physicochemical properties, separations (solid–liquid and liquid–liquid extractions), and drug delivery systems, among others, thus reflecting the disparate interests of the scientific community active in this field.

The articles described in this Special Issue are as follows: Reconcentrating the ionic liquid EMIM-HSO_4_ using direct contact membrane distillation (Contribution 1); determination of enthalpies of vaporization for molecular and ionic liquids using vapor pressure measurements, including quartz crystals, thermogravimetric analysis, and gas chromatographic methods (Contribution 2); conformational geometry matters: the case of the low-melting-point systems of tetrabutylammonium triflate with fumaric or maleic acid (Contribution 3); selectivities of carbon dioxide over ethane in three methylimidazolium-based ionic liquids: experimental data and modeling (Contribution 4); exploration of the solubility hyperspace of selected active pharmaceutical ingredients (APIs) in choline- and betaine-based deep eutectic solvents: machine learning modeling and experimental validation (Contribution 5); and carbon dioxide solubility in three bis(trifluromethylsulfonyl) imide-based ionic liquids to mitigate carbon dioxide emission in Earth’s atmosphere (Contribution 6). These articles examine the physicochemical properties of disparate ILs and DESs, including enthalpies of vaporization, solubility and selectivities of CO_2_ and ethane, and solubility of APIs. Some of them also include machine learning modeling to explain experimental data. Another group of articles describes tuning ferulic acid solubility in choline–chloride- and betaine-based deep eutectic solvents: experimental determination and machine learning modeling (Contribution 7); synergistic solvent extraction of lanthanoids with traditional ligands (4-acylpyrazolone and bidentate nitrogen bases) in a nontraditional diluent confirmed by slope analysis and NMR (Contribution 8); recovery of metals such as cobalt, nickel, copper, lithium, and manganese from the “black mass” of waste portable Li-ion batteries with choline–chloride-based deep eutectic solvents and bi-functional ionic liquids by solid–liquid extraction (Contribution 9); highly efficient and selective extraction of gold from thiosulfate leaching solution using functionalized dicationic ionic liquids (Contribution 10); and study of Aliquat 336 in liquid–liquid extraction chemistry of ReO_4_^−^ anions, including diluents such as carbon tetrachloride, chloroform, and cyclohexane (Contribution 11). These articles describe the excellent properties of ILs and DESs for the extraction of gold, other metal ions from waste batteries, lanthanoids, and ReO_4_^−^. Magnetic ionic liquids (MILs) are a unique class of materials that combine the desirable properties of ionic liquids with intrinsic magnetic behavior. This combination enables a range of innovative applications in catalysis, separation, electrochemistry, biomedicine, and environmental remediation. Given their multifunctional nature, MILs have gained significant attention in recent years for developing smart, efficient, and green technologies. In this issue, one article describes MIL is a multifunctional platform for the design of hybrid graphene/carbon nanotube networks as electromagnetic wave-absorbing materials (Contribution 12). Apart from their use in innovative solvents, both ILs and DESs find exponential increases in APIs; antimicrobial, antifungal, and anticancer applications; and even in drug delivery systems (DDSs), which are wonderful applications of these materials to the global community. Compared to conventional systems delivering antibiotics without a polymer carrier, the choline-based polymers in DDSs attained satisfactory levels of drug loading content and co-release from the polymer carriers, offering a promising alternative for antibiotic delivery (Contribution 13). Among the many potential applications of ILs and DESs, the most significant one is their use in energy storage systems. The search for clean and sustainable energy has become one of the most important research challenges of this century, and they are considered a class of electrolytes with the most potential for the creation of more advanced and safer energy storage systems, including batteries, supercapacitors, and fuel cells. Non-substituted imidazolium-based electrolytes as potential alternatives to the conventional acidic electrolytes of polyaniline-based (PANI) electrode materials for supercapacitors. These electrolytes could enhance the electrochemical performances of PANI electrodes and could be a good alternative to the conventional electrolytes for supercapacitors (Contribution 14). Green synthesis involves the one-pot synthesis and antimicrobial activity of 1,4-dihydropyridine derivatives in aqueous micellar solution under microwave irradiation (Contribution 15). Another green synthesis is the preparation, characterization, and biological activities of an oil-in-water nanoemulsion from fish by-products and lemon oil by the ultrasonication method (Contribution 16).

Apart from the 16 articles, this Special Issue also includes six excellent reviews written by experts in this important field. They are as follows. Ionic liquids as starch plasticizers: the state of the art (Contribution 17); research progress in ionic liquid-based electrolytes for electrochromic devices (Contribution 18); the role of ionic liquids in textile processes: a comprehensive review (Contribution 19); computational advances in ionic liquid applications for green chemistry: a critical review of lignin processing and machine learning approaches (Contribution 20); new materials for thin-film solid-phase microextraction (TF-SPME) and their use for isolation and preconcentration of selected compounds from aqueous, biological, and food matrices (Contribution 21); and ionic liquid-catalyzed CO_2_ conversion for valuable chemicals (Contribution 22).

In conclusion, we believe that this Special Issue, “Advances of Ionic Liquids in Organic, Polymer and Material Chemistry: Key Trends in Green Chemistry”, touches on the latest advancements in many aspects related to ILs and DESs, including their use in electrochromic devices, starch composites for biodegradable materials, even computational advances in IL applications for green chemistry, and conversion of CO_2_ to valuable products to combat CO_2_ emissions. We wish to express our deepest and sincere gratitude to all authors who contributed for having submitted manuscripts of such excellent quality. We also wish to thank the Editorial Office of *Molecules*, especially Ms. Hanna Xiong, for the fast and professional handling of the manuscripts during the whole submission process and for the invaluable help provided to us.
